# A Novel Bioimpedance-Based Detection of Miltefosine Susceptibility Among Clinical *Leishmania donovani* Isolates of the Indian Subcontinent Exhibiting Resistance to Multiple Drugs

**DOI:** 10.3389/fcimb.2021.768830

**Published:** 2021-11-29

**Authors:** Souradeepa Ghosh, Souvik Biswas, Sandip Mukherjee, Arijit Pal, Aaditya Saxena, Shyam Sundar, Jean-Claude Dujardin, Soumen Das, Syamal Roy, Rupkatha Mukhopadhyay, Budhaditya Mukherjee

**Affiliations:** ^1^ School of Medical Science and Technology, Indian Institute of Technology, Kharagpur, India; ^2^ Department of Infectious Disease and Immunology, Council of Scientific & Industrial Research (CSIR)-Indian Institute of Chemical Biology, Kolkata, India; ^3^ Department of Medicine, Institute of Medical Sciences, Banaras Hindu University, Varanasi, India; ^4^ Molecular Parasitology, Institute of Tropical Medicine, Antwerp, Belgium; ^5^ Department of Biomedical Sciences, University of Antwerp, Antwerp, Belgium

**Keywords:** cross-resistance, antileishmanial drug, dose–response analysis, non-protein thiol, bioimpedance

## Abstract

The extent of susceptibility towards miltefosine (Mil), amphotericin B (AmpB), and paromomycin (Paro) was measured among 19 clinical isolates of *Leishmania donovani* (LD). Thirteen of these clinical isolates were reported to exhibit low susceptibility towards sodium stibogluconate (SSG-R), while six of them were highly susceptible (SSG-S). The degree of clearance of amastigotes (EC50) for these predefined SSG-R- and SSG-S-infected macrophages was determined against Mil, AmpB, and Paro. Two out of the 13 SSG-R isolates (BHU575 and BHU814) showed low susceptibility towards all three drugs studied, while the rest of the 11 SSG-R isolates showed varying degrees of susceptibility either towards none or only towards individual drugs. Interestingly, all the SSG-S isolates showed high susceptibility towards Mil/AmpB/Paro. The total intracellular non-protein thiol content of the LD promastigotes, which have been previously reported to be positively co-related with EC50 towards SSG, was found to be independent from the degree of susceptibility towards Mil/AmpB/Paro. Impedance spectra analysis, which quantifies membrane resistance, revealed lower impedimetric values for all those isolates exhibiting low efficacy to Mil (Mil-R). Our analysis points out that while non-protein thiol content can be an attribute of SSG-R, lower impedimetric values can be linked with lower Mil susceptibility, although neither of these parameters seems to get influenced by the degree of susceptibility towards AmpB/Paro. Finally, a correlation analysis with established biological methods suggests that impedance spectral analysis can be used for the accurate determination of lower Mil susceptibility among LD isolates, which is further validated in the LD-infected *in vivo* hamster model.

## Introduction

Visceral leishmaniasis is caused by the parasites of *Leishmania donovani* (LD) complex and is fatal if left untreated. Since there is a limited armory of drugs and protective vaccines against leishmaniasis to date, chemotherapy is the only remaining option. Pentavalent antimony has been the mainstay of leishmaniasis chemotherapy for almost a century, which not only showed serious side effects but also has been gradually losing its efficacy in some regions of India due to increasing parasite resistance (Dumetz et al., 2018). Around 78% of LD clinical isolates prevailing in the Indian subcontinent have been reported to show low susceptibility to antimonial drugs (Mukhopadhyay et al., 2011). Several incidences of antimony resistance in India and all over the world have been marked by relapse cases in patients (Hefnawy et al., 2017; Ponte-Sucre et al., 2017). Current treatment options include the polyene antibiotic amphotericin B (AmpB) (deoxycholate liposomal complex ambisome), the aminoglycoside antibiotic paromomycin (Paro), and the alkylphosphocholine miltefosine (Mil). Short treatment courses that include combination therapy by the co-administration of two drugs are also effective (van Griensven et al., 2010; Mahajan et al., 2015; Goswami et al., 2020; Mukherjee et al., 2020).

Previous studies have reported that sodium antimony gluconate-resistant (SSG-R) field isolates have started exhibiting *cross-resistance to AmpB and Mil, with overexpression of HSP70 and HSP83 being implicated in conferring SSG and AmpB resistance* (García-Hernández et al., 2012; Prasanna and Upadhyay, 2021). In contrast, reports suggest that HSP70 is downregulated in case of miltefosine-resistant LD (Mil-R), which may have an indirect anti-apoptotic effect (Vacchina et al., 2016). Some of the drugs, such as Mil, are available over the counter, and since it is known to have a prolonged half-life, the probability of resistance development against Mil is quite high due to improper use (Sundar and Olliaro, 2007; Dorlo et al., 2014; Ramesh et al., 2015). It is also probable that resistance against Paro, which is an aminoglycoside, will emerge rapidly (Jhingran et al., 2009; Bhandari et al., 2014). Although several mechanisms are leading to resistance development against these antileishmanial drugs, there may be a common factor that leads to the emergence of cross-resistance.

Here we report on the efficacy of the three commonly used antileishmanial drugs (Mil, Paro, and AmpB) on an *in vitro* peritoneal macrophage model infected with previously characterized SSG-R and SSG-S clinical isolates from highly resistant zones of Bihar. Based on the activity of Mil, AmpB, and Paro on these isolates, we can distinctly identify two clinical isolates (BHU575 and BHU814) with high EC_50_ values against all three drugs. Furthermore, we have standardized a novel impedance-based spectral analysis that can be used to identify LD promastigotes showing a lower efficacy towards Mil, probably owing to its differential membrane composition, which will open a window for their clinical identification through further biochemical characterization of these Mil-R isolates.

## Materials and Methods

### Animal and Ethics Statement

BALB/c mice (*Mus musculus*) and golden hamsters (*Mesocricetus auratus*) were maintained and bred under pathogen-free conditions. The use of both mice and hamsters was approved by the Institutional Animal Ethics Committees of Indian Institute of Chemical Biology, India. All animal experiments were performed according to the National Regulatory Guidelines issued by CPSEA, IAEC (Committee for the Purpose of Supervision of Experiments on Animals, IICB/AEC-15-2008, 10.06.2008; biosafety clearance number, IITKGP RCGM: BT/BS/17/27/97-PID, 11.02.21).

### Isolation of Macrophages

Mouse peritoneal macrophages (PECs) were harvested from BALB/c mice by lavage 48 h after an intraperitoneal injection of 2% soluble starch (Sigma). For convenience, the term PECs and macrophages are used interchangeably. The macrophages were plated on sterile 22-mm^2^ coverslips (Bluestar) in 35-mm disposable petri plates (Tarsons) at a density of 1 × 10^5^ cells/coverslip in RPMI 1640 medium (Sigma) supplemented with 10% fetal bovine serum (FBS; Sigma) and 100 U PenStrep (Gibco) (RPMI complete medium). The cells were left to adhere for 48 h at 37°C and 5% CO_2_ before infection.

### Parasites and Infection

Promastigotes of 13 and six previously characterized clinical *L. donovani* isolates showing resistance or susceptibility to antimonial drugs, respectively, were maintained in M-199 complete medium. The respective years of isolation of the parasites and the treatments received by the patients are mentioned previously (Mukhopadhyay et al., 2011). Furthermore, all the clinical LD isolates used in this study were clonal isolates whose EC50 against SSG was determined and matched with a previously reported EC50 value. The host cells (PECs) were infected with metacyclic promastigotes at a ratio of 1:10 as described previously (Mukhopadhyay et al., 2011). Briefly, all experimental infections were performed with stationary-phase early-passage promastigotes. Similarly, for impedance and thiol measurement, early-passage (passage 2–5) stationary-phase promastigotes were used as described previously (Mukhopadhyay et al., 2011). All promastigotes were checked for their EC_50_ against SSG, and isolates showing comparable EC_50_ values with no significant difference from their previously reported susceptibility levels towards SSG (Mukhopadhyay et al., 2011) were further characterized against Mil, AmpB, and Paro.

### Preparation of Drug Stocks and Drug Assays

A 2-mM stock of Amp-B (SIGMA) was prepared in dimethyl sulfoxide and subjected to sterile filtration. Mil (received through Kaladrug-R consortium) and Paro sulfate (Sigma) stock solutions were prepared at 20 mM in water and filter-sterilized. SSG (Albert David, Kolkata) was prepared at 1 mg/ml of 30.30% Sb^V^ in 1X phosphate-buffered saline (PBS); and dissolution of the powder was being aided by incubation at 37°C overnight, and the solution was then subjected to sterile filtration. All aqueous drug solutions appeared clear and fully dissolved.

The drugs were threefold serially diluted over six concentrations in RPMI 1640 medium supplemented with 10% FBS and 100 U PenStrep in triplicate at each concentration. Stock solutions and dilutions were prepared fresh for each use. The infected macrophages were incubated with drug dilutions for a total of 48 h at 37°C and 5% CO_2_. The untreated macrophages received RPMI complete medium, and infection was determined at 48 h post-treatment. Before the experiment, the dose of each drug that was tolerated by the macrophage was determined as described previously (Mukhopadhyay et al., 2011). At the endpoints, the coverslips were washed with PBS, dried and fixed with 100% methanol (Merck), stained with 10% Giemsa (SIGMA), and examined microscopically. The EC_50_ values of each isolate were estimated (Kremb et al., 2010; Mukhopadhyay et al., 2011) against each drug by the method recommended by the Kaladrug-R consortium. The experiment was repeated thrice.

### Cell Viability Assay

The dose of each drug that was tolerated by the macrophage was already determined. Briefly, uninfected PEC (0.5 × 10^6^) cells were treated with each of the dilutions of the four drugs for a total of 48 h at 37°C and 5% CO_2_. The percentage of cell viability was checked 48 h later by trypan- Blue staining and counting under a light microscope. The cells were washed with PBS and incubated with 0.4% Trypan blue (Thermo Fisher Scientific) solution in PBS for 15 min (Strober, 2015). The concentration ranges for the experimental sets were adjusted to cover EC_50_ values where possible. In parallel experiments, parasites were incubated with different doses of Mil (μM), and then their susceptibility to the drug was observed by MTT (Sigma) and Alamar Blue (ThermoFisher) assay according to the protocol of the manufacturers.

### Determination of Intracellular Non-protein Thiol in the Clonal Populations

Two million stationary-phase parasites were used for the study. Each of the samples was washed with sterile PBS, incubated with 5 μM of 5-chloromethylfluorescein-diacetate (Molecular Probes, Carlsbad, CA, USA), incubated for 15 min at 35°C in PBS in the dark as described elsewhere, and subjected to flow cytometric analysis (Sarkar et al., 2009).

### Determination of Electrical Bioimpedance Spectroscopy for LD Promastigotes

The impedance measurement of the parasites suspended in PBS was carried out in an ECIS-8W1E DD (Applied BioPhysics, USA) cell culture well. An Agilent precision impedance analyzer 4294-A, in the frequency range of 40 Hz to 100 MHz, was used to record the impedance data from the ECIS device by applying 10-mV AC excitation voltage. The ECIS-8W1E DD culture well consists of eight separate mini-culture wells having an individual circular working electrode and common counter-electrode made of a thin gold film as described previously (Das et al., 2012). The impedimetric responses were acquired with 400 µl of PBS for measuring baseline and subsequently 400 µl of PBS containing 10^6^ promastigotes to study the activity of LD isolates.

### Infection of Hamsters and Determination of Splenic and Liver Parasite Burden With and Without Mil Treatment

Promastigotes of LD strains BHU782, BHU814, BHU777, and BHU816 were used to infect 4- to 6-week-old golden hamsters. At 45 days post-infection, 17.5 mg/kg Mil was administered orally for 30 days. A human-equivalent dose of Mil for the hamster was used as described elsewhere (Reagan-Shaw et al., 2008; Fortin et al., 2016). At 7 days post-completion of treatment, splenic and liver parasite burden was determined by the stamp–smear method as described previously (Basu et al., 2005).

### Statistical Analysis

Each experiment was performed three times independently in duplicates, and the results are expressed as mean ± SD. Statistical significance was calculated by implementing *t*-test, and a P-value <0.05 was considered statistically significant. Values were considered most significant (represented as ****) if *p <*0.0001, extremely significant (represented as ***) if *p <*0.001, very significant (represented as **) if *p* =0.001–0.01, or significant (represented as *) if *p* =0.01–0.05; non-significance was represented as “ns”. To establish a correlation between the EC_50_ with other parameters, the Spearman rank correlation coefficient was used and expressed as *r*. Data were analyzed using Graphpad Prism software.

## Results

### Susceptibility Assay of the Intracellular SSG-R LD to Three Antileishmanial Drugs (Paro, Mil, and AmpB)

The previously characterized clonal population of field isolates, obtained from patients in the SSG-unresponsive zone, was subjected to test for susceptibility to three different antileishmanial drugs at intracellular amastigote stages (Seifert et al., 2010). The EC_50_ values of different isolates at their intracellular amastigote stages are shown in **Table 1**. The EC_50_ values for AmpB at the amastigote stage ranged from 0.01 ± 0.004 to 0.368 ± 0.199 µM, with a mean EC_50_ of 0.189 ± 0.10 µM. The field isolates showed various levels of susceptibility to Mil, with EC_50_ values at the amastigote stage ranging from 0.04 ± 0.026 to 6.13 ± 0.315 µM, with a mean EC_50_ of 3.085 ± 0.17 µM. For Paro, the EC_50_ values ranged from 1.01 ± 0.168 to 96.95 ± 16.96 µM, with a mean EC_50_ of 48.98 ± 8.56. This indicates a very high variation of field resistance among different isolates with respect to all three drugs. The antileishmanial activity of SSG indicates a medium positive correlation (*r* = 0.407, *p* < 0.001 and *r* = 0.431, *p* < 0.001) with Paro and Mil, respectively, and low positive correlation (*r* = 0.119, *p* < 0.001) with AmpB. Among 13 previously characterized SSG-R LD, two (BHU575 and BHU814) showed high EC_50_ against all three anti-leishmanial drugs, while BHU770, BHU872, and BHU782 showed high EC_50_ against Mil and Paro. BHU744 showed high EC_50_ against Mil, while BHU796 and BHU569 showed high EC_50_ against Paro and AmpB, respectively. Interestingly, some SSG-R LD (BHU568, BHU764, BHU572, BHU573, and BHU574) showed low EC_50_ to all the three drugs. However, all previously characterized SSG-S LD (BHU816, BHU777, BHU581, BHU741, Ag83, and BPK206) showed low EC_50_ to all three drugs as presented in **Table 1**.

**Table 1:** Susceptibility of intracellular amastigotes (EC_50_) to different antileishmanial drugs.

Sl. No.	Groups	Isolate Code	(μM)		
			Miltefosine	Paromomycin	Amphotericin B
1	1. SSG-R/Mil-R/ Paro-R/AmpB-R	BHU 575	3.45 ± 0.28	50.79 ± 2.53	0.35 ± 0.13
2		BHU 814	6.14 ± 0.32	103.69 ± 15.79	0.47 ± 0.34
3	2. SSG-R/Mil-R, Paro-R	BHU 770	3.37 ± 0.87	57.81 ± 9.28	0.01 ± 0.01
4		BHU 872	6.14 ± 0.05	79.06 ± 11.79	0.11 ± 0.02
5		BHU 782	6.14 ± 0.69	53.09 ± 8.29	0.16 ± 0.07
6	3. SSG-R/Mil-R	BHU 744	5.64 ± 1.16	21.57 ± 4.41	0.02 ± 0.01
7	4. SSG-R/Paro-R	BHU 796	1.23 ± 0.17	96.95 ± 16.96	0.01 ± 0.01
8	5. SSG-R/AmpB-R	BHU 569	0.05 ± 0.02	8.09 ± 1.84	0.40 ± 0.02
9	6. SSG-R	BHU 568	0.07 ± 0.02	1.01 ± 0.17	0.09 ± 0.06
10		BHU 764	0.03 ± 0.01	1.24 ± 0.87	0.01 ± 0.01
11		BHU 572	0.15 ± 0.02	2.69 ± 2.44	0.02 ± 0.01
12		BHU 573	2.46 ± 0.49	6.74 ± 1.14	0.04 ± 0.02
13		BHU 574	0.05 ± 0.03	5.39 ± 1.6	0.03 ± 0.01
14	7. SSG-S/Mil-S, Paro-S/AmpB-S	BHU 816	0.17 ± 0.08	39.92 ± 12.56	0.02 ± 0.01
15		BHU 777	0.25 ± 0.15	1.13 ± 1.11	0.02 ± 0.01
16		BHU 581	0.25 ± 0.03	8.09 ± 1.39	0.09 ± 0.02
17		BHU 741	1.84 ± 0.49	0.20 ± 0.13	0.01 ± 0.01
18		Ag 83	0.05 ± 0.03	1.35 ± 0.70	0.01 ± 0.00
19		BPK 206	0.12 ± 0.10	11.59 ± 6.2	0.07 ± 0.03

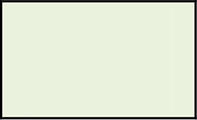
 Low susceptibility towards SSG, Mil, Paro, and AmpB.

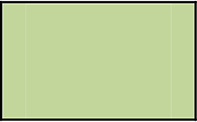
 Low susceptibility towards SSG, Mil, and Paro.

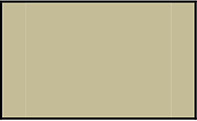
 Low susceptibility towards SSG and Mil.

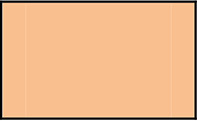
 Low susceptibility towards SSG and Paro.

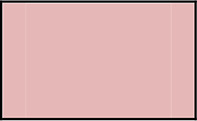
 Low susceptibility towards SSG and AmpB.

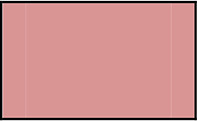
 Low susceptibility towards SSG.

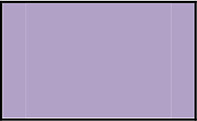
 High susceptibility towards SSG, Mil, Paro, and AmpB.

### *In Vitro* Susceptibility of the LD Promastigotes to Mil Determined by MTT and Alamar Blue Assay

For a comparative evaluation of IC_50_ and EC_50_ against Mil, two different dyes (MTT and Alamar Blue) were used. The MTT and Alamar Blue results show that the isolates having high EC50 against Mil in amastigote stage also showed the highest IC_50_ against MIL in MTT and Alamar Blue assay. Those resistant to SSG alone show moderate IC_50_ against MIL, while SSG-sensitive isolates show low IC_50_ to MIL (**Table 2**).

**Table 2:** Determination of IC_50_ against Miltefosine using MTT and Alamar Blue cell viability assay.

Sl. No.	Groups	Isolate code	Alamar blue (IC_50_ in mM)	MTT (IC_50_ in mM)
1	1. SSG-R/Mil-R/ Paro-R/AmpB-R	BHU 575	8.8 ± 1.4	9.7 ± 0.5
2		BHU 814	13.1 ± 0.8	13.7 ± 0.5
3	2. SSG-R/Mil-R, Paro-R	BHU 770	8.4 ± 1.3	8.0 ± 0.4
4		BHU 872	17.8 ± 0.8	17.2 ± 0.9
5		BHU 782	16.5 ± 1.4	16.7 ± 1.4
6	3. SSG-R/Mil-R	BHU 744	12.6 ± 0.9	12.5 ± 1.03
7	4. SSG-R/Paro-R	BHU 796	7.2 ± 1.2	6.9 ± 1.9
8	5. SSG-R/ AmpB-R	BHU 569	5.7 ± 0.6	4.1 ± 0.9
9	6. SSG-R	BHU 568	3.7 ± 0.3	3.7 ± 0.2
10		BHU 764	3.5 ± 0.4	2.4 ± 0.3
11		BHU 572	3.3 ± 0.3	2.7 ± 0.2
12		BHU 573	7.1 ± 1.4	6.5 ± 1.7
13		BHU 574	12.4 ± 0.7	11.6 ± 0.6
14	7. SSG-S/Mil-S, Paro-S/AmpB-S	BHU 816	2.9 ± 0.2	2.8 ± 0.6
15		BHU 777	3.1 ± 0.8	3.1 ± 0.9
16		BHU 581	5.9 ± 0.8	4.5 ± 0.3
17		BHU 741	5.5 ± 0.3	6.1 ± 0.5
18		Ag 83	4.5± 0.8	5.1± 0.4
19		BPK 206	7.3 ± 0.7	6.5 ± 0.5

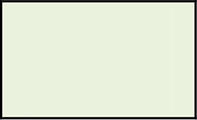
 Low susceptibility towards SSG, Mil, Paro, and AmpB.

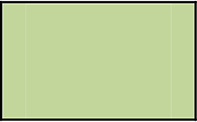
 Low susceptibility towards SSG, Mil, and Paro.

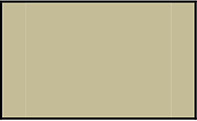
 Low susceptibility towards SSG and Mil.

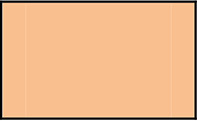
 Low susceptibility towards SSG and Paro.

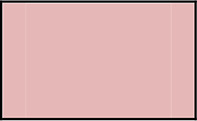
 Low susceptibility towards SSG and AmpB.

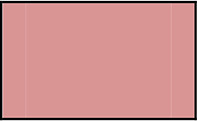
 Low susceptibility towards SSG.

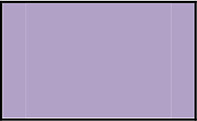
 High susceptibility towards SSG, Mil, Paro, and AmpB.

### Intracellular Thiol Content Can Only Distinguish Between Clonal LD Populations Based on Their Susceptibility Towards SSG

**Figure 1:**
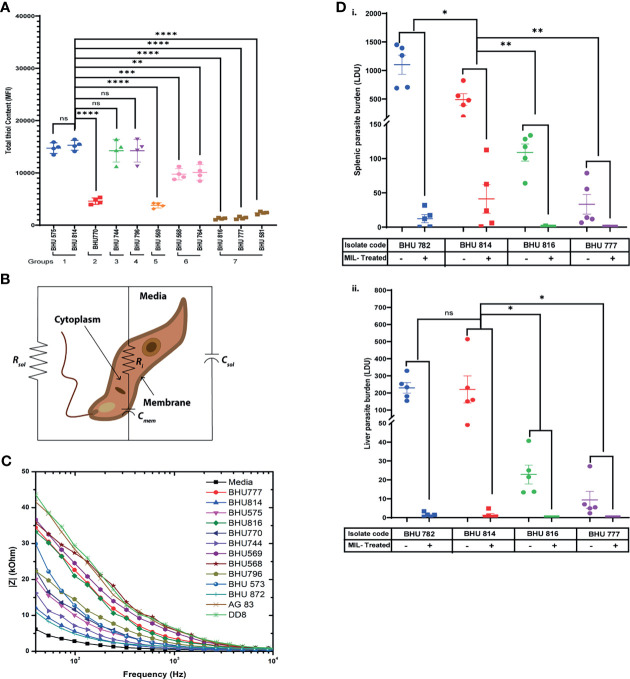
**(A)** Flow cytometric detection of intracellular thiol content in both SSG-R and SSG-S isolates using the fluorescence probe 5-chloromethylfluorescein-diacetate (CMFDA). Two million stationary-phase parasites were incubated in 5 μM of CMFDA in phosphate-buffered saline (PBS) at 37°C for 15 min. Fluorescence was measured in log mode and expressed as mean fluorescence intensity (MFI). Data are expressed as MFI ± SD for three independent experiments. ****p < 0.0001, ***p < 0.001, **p = 0.001–0.01, *p = 0.01–0.05; ns, non-significance. **(B)** Electrical equivalent circuit model for *Leishmania donovani* (LD) promastigotes suspended in media. *R_i_* and *C*_mem_ denote cytoplasm resistance and membrane capacitance, respectively, which may vary depending upon cell types. Solution resistance (*R*_sol_) and capacitance (*C*_sol_) are present due to the ionic conductivity of PBS and electrode–electrolyte interface. **(C)** Impedance spectrum showing the magnitude of impedance *vs.* frequency (Hz) for LD isolates. The magnitude of impedance was recorded against a frequency range of 40 Hz to 100 MHz, with a sinusoidal excitation voltage of 10 mV. A significant change is visible within the frequency range of 40 Hz to 10 kHz as depicted in the figure. Representative figure is presented from three independent experiment. **(D)** Splenic and hepatic parasite burden of hamsters infected with either BHU782 and BHU814 isolates (showing low susceptibility towards Mil) or BHU816 and BHU777 (showing high susceptibility towards Mil) and either treated with 17.5 mg/kg Mil or left untreated was expressed as Leishman Donovan units, calculated as the number of parasites per 1,000 nucleated cells  ×  weight of the organ (in mg). Data are expressed as means ± SD, with statistical significance being determined for parasite clearance after Mil treatment comparing Mil less susceptible strains (BHU 782 and BHU 814) with highly susceptible strains (BHU 816 and BHU 777). **p = 0.001–0.01; ns, non-significance.

It has been well established that SSG-R isolates have a higher content of intracellular thiols than the sensitive strains (Rai et al., 2013). However, whether these higher thiol contents can also influence resistance to other antileishmanial drugs is yet to be defined. To evaluate this based on EC_50_ data, the LD isolates were grouped in seven distinct categories, and intracellular thiol content was determined for each representative group: group 1 representing high EC_50_ against all drugs; group 2 with moderate EC_50_ values against SSG, along with high resistance towards Mil/Paro; group 3 representing high EC_50_ values against SSG and Mil; group 4 with high EC_50_ values against SSG and Paro; group 5 with high EC_50_ values against SSG and AmpB; group 6 having high EC_50_ against only SSG; and group 7 was representative SSG-LD with low EC_50_ against all other drugs. Our analysis suggests that isolates with high EC_50_ against SSG tend to have a significantly higher intracellular non-protein thiol as compared to those isolates having low EC_50_ against SSG and are not affected by EC_50_ against any of the other three drugs, although it is enhanced in case of high EC_50_ against all the drugs mentioned (**Figure 1A**)—for instance, the level of non-protein thiol is significantly higher in BHU568 which shows high EC_50_ against only SSG and low EC_50_ against all other drugs. Thiol content is highest in BHU814 with high EC_50_ against SSG, Mil, and Paro in contrast to BHU770 with lower thiol content and having moderate EC_50_ against SSG but high EC_50_ against both Mil and Paro. Similarly, BHU569, which exhibits a high EC_50_ against AmpB, exhibits much lower non-protein thiol content owing to its moderate and low EC_50_ against SSG, respectively. Taken together, our data suggest that isolates with high EC_50_ against SSG independent of EC_50_ values of all other drugs tend to have a high non-protein thiol content in contrast to isolates with lower (as in the case of SSG-S isolates BHU816 and BHU777) EC_50_ against SSG.

### Impedance Spectral Analysis Can Successfully Distinguish Between Mil-Resistant and Sensitive Clonal LD Populations

Previously, fluorescence anisotropy has been used to characterize between SSG-S and SSG-R isolates, with SSG-R parasites showing higher membrane fluidity (Mukhopadhyay et al., 2011). As intracellular non-protein thiol content can distinguish LD isolates with only high EC_50_ against SSG but fails to distinguish between other drugs used in this study, we decided to check whether we can use membrane fluidity as a possible parameter to distinguish between LD isolates showing varying degrees of susceptibility towards Mil, Amp B, and Paro. By using impedance-based spectral analysis, we determine resistance and capacitance for members of group 1 (BHU575 and BHU 814), group 2 (BHU770 and BHU 872), group 3 (BHU744), group 4 (BHU796), group 5 (BHU569), group 6 (BHU 568 and BHU 573), and group 7 (BHU816, BHU777, Ag83, and DD8). These parameters are then fitted into a Bode plot using an equivalent circuit model (**Figure 1B**) in the EC-lab software to extract the resistance and capacitance of cell membrane (Das et al., 2014). The equation is given as follows: overall impedance =* R*_sol_ || *C*_sol_ || (*R_i_* +*C*_mem_), where *R*_sol_ and *C*_sol_ are solution resistance and solution capacitance respectively. *R_i_* represents cytoplasm resistance, and *C*_mem_ represents membrane capacitance. Here changes in *R_i_* and *C*_mem_ are mainly considered for analysis, as solution resistance (*R*_sol_) and capacitance (*C*_sol_) do not affect the overall impedance. *R*_sol_ and *C*_sol_ depend on the conductivity of the media solution where the parasites were suspended. In case of media, as no cells are present in the solution, the overall equivalent circuit becomes *R*_sol_ || *C*_sol_. A lower value of *R*_sol_ confirms the higher conductivity of media, as it contains more number of ions. *R*_sol_ in LD strains increases significantly, as the incubated parasites produce insulating byproducts, and thus drastically reduces the conductivity of the solution. *C*_sol_, in case of media, will not vary significantly, as it is present due to the electrode–electrolyte interface. Our result suggests that SSG-R isolates having a high EC_50_ value only against Mil, irrespective of their susceptibility towards other drugs, exhibited a significantly higher membrane fluidity owing to its higher membrane capacitance (**Figure 1C**). This is particularly evident for BHU814, BHU 872, BHU744, BHU575, and BHU 770 which have higher EC_50_ towards Mil and showed a higher value for *C*_mem_ (nF), decreasing the overall impedance denoted by |Z| (kOhm), as shown in **Figure 1C**; however, cytoplasmic resistance (*Ri*), despite being the variable, does not change much in different parasites (**Table 3**).

**Table 3:** Determination of different impedimetric parameters for LD promastigotes suspended in media.

Sl. No.	Groups	Strains	Electrical Parameters			
			*R_sol_* (Ohm)	*R_i_* (Ohm)	*C_mem_* (nF)	*C_sol_* (pF)
1	1. SSG-R/Mil-R/ Paro-R/AmpB-R	BHU575	45645	220.8	125.6	33.43
2		BHU814	31764	213.6	255.4	34.71
3	2. SSG-R/Mil-R, Paro-R	BHU770	49169	232.3	119.5	29.64
4		BHU 872	8387	245.2	258.9	6.18
5	3. SSG-R/Mil-R	BHU744	32260	219.6	185.6	39.38
6	4. SSG-R/Paro-R	BHU796	33419	226.7	90.38	23.51
7	5. SSG-R/ AmpB-R	BHU569	42245	208	44.69	4.991
8	6. SSG-R	BHU568	33816	204.9	34.89	7.039
9		BHU 573	97 096	160.8	111.6	11.53
10	7. SSG-S/Mil-S, Paro-S/AmpB-S	BHU816	53967	215.8	62.37	24.11
11		BHU777	38350	229.9	47.86	18.59
12		Ag 83	61748	241.1	42.23	15.28
13		DD8	87009	248.5	57.5	11.2

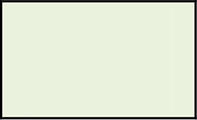
 Low susceptibility towards SSG, Mil, Paro, and AmpB.

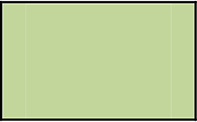
 Low susceptibility towards SSG, Mil, and Paro.

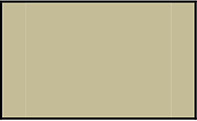
 Low susceptibility towards SSG and Mil.

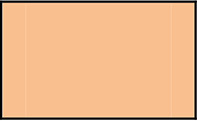
 Low susceptibility towards SSG and Paro.

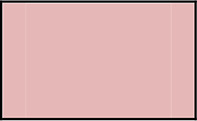
 Low susceptibility towards SSG and AmpB.

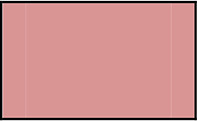
 Low susceptibility towards SSG.

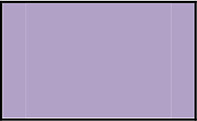
 High susceptibility towards SSG, Mil, Paro, and AmpB.

### Correlation Analysis Between EC_50_ and IC_50_ Determined by Alamar Blue/MTT, With Impedance Spectral Analysis for Low Mil-Susceptible LD Isolates

To determine whether impedance-based spectral analysis can be used to determine low Mil susceptibility among clinical LD isolates, a correlation analysis was performed. It is evident from our analysis that, while EC_50_ values show a positive correlation with IC_50_ values against Mil-R LD isolates, a significant correlation of EC_50_ was also observed with *C*_mem_ (*r* = 0.96716, *p* < 0.001) and *C*_sol_ (*r* = 0.85427, *p* < 0.001). Hence, our result suggests that impedimetric parameters can be used to detect low miltefosine susceptibility in LD isolates.

### Effect of Mil Treatment on Organ Parasite Burden in Hamsters Infected With Promastigotes

The results of our previous drug sensitivity and bioimpedance analysis implied that there are six LD isolates (BHU575, BHU814, BHU770, BHU872, and BHU782) with low sensitivity to Mil and BHU816, BHU777, BHU581, BHU741, Ag83, and BPK206 show high sensitivity towards Mil. Out of these, two isolates (BHU814 and BHU782) that are less sensitive to Mil and two isolates (BHU777 and BHU816) showing high sensitivity towards Mil were used to infect golden hamsters. After the indicated time of Mil treatment, the infected hamsters and both the Mil-treated and untreated groups were sacrificed, and splenic and liver parasite number was determined. There were no detectable parasites in either the spleen or liver of the hamsters infected with BHU777 or BHU816 after Mil treatment; however, a significant number of residual parasites were observed in the spleen and liver of hamsters infected with BHU782 or BHU814 even after 1 month of treatment with Mil (seven times higher than the human-equivalent dose in hamsters), as shown in **Figure 1D** (i and ii), suggesting that these isolates do exhibit low susceptibility towards Mil *in vivo*.

## Discussion

From our results on the efficacy of the four antileishmanial drugs on an *in vitro*-infected murine macrophage model, it is clear that isolates BHU575, BHU814, BHU770, BHU872, BHU782, BHU744, BHU796, and BHU569 show a high EC_50_ value against more than one drug, including SSG (*i*.*e*., AmpB, Mil, and Paro). Furthermore, it is interesting to note that isolates (BHU816 and BHU777) having lower EC_50_ against SSG also showed sensitivity to all the other three common anti-leishmanial drugs. This prompts us to speculate that antimony resistance is probably the initial ignition that triggers cross-resistance against other drugs. Although normal macrophages can sustain up to 18.18 μg/ml of SSG, their SSG tolerance is increased upon infection of the macrophage with SSG-resistant parasite, which may be due to the efflux of antimony from the cells (Mukhopadhyay et al., 2011). Previously, it has been shown that macrophages infected with SSG-resistant parasites show an upregulation of ABC transporters, which favors antimony efflux. Based on our observation, the SSG tolerance level in macrophages appears to become higher upon infection with SSG-resistant isolates. This may be because the drug is effluxed from the cell as the amastigotes take control of the host cell machinery and induce the overexpression of ABC transporters on the host surface (Mookerjee Basu et al., 2008; Rai et al., 2013; Perea et al., 2016). There are a few reports of cross-resistance in field isolates where it is suggested that all the drugs may have a common way of parasite killing, like programmed cell death, although the mechanism might be different (Vergnes et al., 2007). In view of the rampant SSG resistance among clinical LD isolates, it is not unlikely that such resistance may have some direct or indirect bearing on the efficacy of other common antileishmanial drugs. The above-mentioned study also supports such similar conclusion which seems to suggest that existing low susceptibility towards SSG has aggravated the development of cross-resistance towards common anti-leishmanial drugs in the Indian subcontinent.

Although the emergence of cross-resistance is a multi-hit process that includes complex molecular as well as biochemical events, in this report, we tried to address the common factor that may lead to the emergence of multi-drug resistance. Cysteine, glutathione, and trypanothione, the thiols occurring in *Leishmania*, are present at an increased level in SSG-R isolates than in the sensitive counterpart (Mandal et al., 2007; Sarkar et al., 2009). A previous study has reported that the thiol content in LD isolates showed a positive correlation with their low SSG susceptibility (Mukhopadhyay et al., 2011). We also find that thiol content is highest in the case of SSG-R isolates with highest EC50 against SSG. However, the degree of susceptibility towards other anti-leishmanial drugs seems to be independent of thiol content. These findings depict that, although SSG-R might trigger cross-resistance depending upon its thiol content to adapt under drug stress, there might be independent additional factors which might govern multi-drug-resistant phenotypes, as thiol content can only distinguish between SSG-R isolates from that of SSG-S LD isolates irrespective of their susceptibility towards other anti-leishmanial drugs. Hence, we further tried to focus on membrane fluidity which has also been previously reported to be higher in SSG-R isolates (Mukhopadhyay et al., 2011). Recently, impedance-based spectral analysis has been shown to correctly determine anti-microbial susceptibility in drug-resistant bacterial pathogens (Spencer et al., 2020). Our impedimetric analysis could only detect the degree of Mil susceptibility among different LD isolates showing differential susceptibility towards different anti-leishmanial drugs, probably owing to the significantly higher membrane fluidity of Mil-R isolates. A complete comparative lipid analysis between different LD isolates showing varying degrees of susceptibility towards different anti-leishmanial drugs would be ideal to address this. Based on our impedimetric analysis, we could successfully distinguish between low and high Mil susceptibility among LD isolates. Our analysis particularly showed a positive correlation between *C*_mem_ and *C*_sol_ determined from impedance spectral analysis with high EC_50_ and IC_50_ values against Mil as determined by a standard biological test. Interestingly, no significant correlation was observed between the *C*_mem_ and *C*_sol_ of LD isolates showing high EC_50_ values against Paro and AmpB.

It is well known that the fatty acid tails of cell membrane phospholipids can either be saturated or unsaturated. In the unsaturated form, the fatty acid tails contain double bonds in carbon atoms, forming a kinked shape that prevents tight packing of the lipids and thus increasing membrane fluidity. Moreover, in terms of current conduction, increasing membrane fluidity allows the electrons to pass freely through the cell membrane. This implies that an increase in membrane fluidity decreases the overall impedance of the cell. Our result suggests that Mil cross-resistant phenotypes have lower impedimetric values, which result in lower membrane resistance and consequently higher membrane fluidity which, in turn, may impart an additional survival advantage to these isolates and need further exploration. This result is supported by previous reports suggesting that the Mil-R LD line possesses higher membrane fluidity, in contrast to all other resistant lines, owing to its high mevalonate content which is the precursor of sterol biosynthesis (Berg et al., 2015). These sterols play a crucial role for maintaining the membrane in a state of fluidity, which is essential for functioning (Dufourc, 2008). In contrast, although AmpB-R LD has also been reported to have higher membrane fluidity, their membrane lipid composition consists mostly of saturated fatty acids, with steric acid being the most prominent one and major sterol being an ergosterol precursor, which should result in a rigid membrane (Mbongo et al., 1998; Purkait et al., 2012). For Paro-R LD, higher membrane fluidity has also been reported, resulting in lower intracellular drug accumulation, although nothing much has been reported on its biochemical composition (Bhandari et al., 2014). Our impedimetric analysis could only detect the degree of Mil susceptibility among different LD isolates showing differential susceptibility towards different anti-leishmanial drugs, probably owing to the significantly higher membrane fluidity of Mil-R isolates. A complete comparative lipid analysis between different LD isolates showing varying degrees of susceptibility towards different anti-leishmanial drugs would be ideal to address this.

To verify that the isolates showing high EC_50_ to Mil also show similar phenotypes *in vivo*, infected hamsters were treated with Mil (17.5 mg/kg/day for 30 days). We found that, at 1 month after Mil treatment, there were residual parasites in the spleen of hamsters infected with Mil-R parasites, whereas Mil-S parasites were completely cleared from the organ. Thus, the results of *in vitro* parasite sensitivity to Mil are also reflected in *in vivo* parasite burden. The question arises as to whether there is a single drug resistance mechanism with a bearing on resistance to other drugs. At this point, it is tempting to speculate that, with the introduction of Mil treatment, tolerance to this drug is increasing, and further characterization of the Mil-resistant parasites is required to address the issue of multi-drug-resistant LD isolates. Since the mechanism of prevalence of resistance to different anti-leishmanial drugs is multifactorial (Hefnawy et al., 2017; Ponte-Sucre et al., 2017), we believe that further in-depth investigations are required for developing a comprehensive biophysical detection approach which could accurately determine susceptibility for other drugs like AmpB and Paro.

## Data Availability Statement

The original contributions presented in the study are included in the article/supplementary material. Further inquiries can be directed to the corresponding authors.

## Ethics Statement

All animal experiments were performed according to the National Regulatory Guidelines issued by CPSEA, IAEC (Committee for the Purpose of Supervision of Experiments on Animals, IICB/AEC-15-2008, 10.06.2008) biosafety clearance number IITKGP RCGM: BT/BS/17/27/97-PID, 11.02.21).

## Author Contributions

Designing and execution of experiments were performed by SG, SB, SM, AP, AS, and RM. Data analysis was carried out by SG, SB, SM, AP, RM, and BM. Preparation of the original draft and editing were done by SG, SB, SM, RM, and BM. SS and JC provide the reagents and materials. SD, SR, RM, and BM participated in supervision and conceptualization. RM and BM conceive the project. SD, SR, and BM secure funding for this work. All authors contributed to the article and approved the submitted version.

## Funding

SG, SB, and AP are recipients of IITKGP-GATE Fellowship. SR is the recipient of JC Bose fellowship.

## Conflict of Interest

The authors declare that the research was conducted in the absence of any commercial or financial relationships that could be construed as a potential conflict of interest.

## Publisher’s Note

All claims expressed in this article are solely those of the authors and do not necessarily represent those of their affiliated organizations, or those of the publisher, the editors and the reviewers. Any product that may be evaluated in this article, or claim that may be made by its manufacturer, is not guaranteed or endorsed by the publisher.
